# Establishment of Real Time Allele Specific Locked Nucleic Acid Quantitative PCR for Detection of HBV YIDD (ATT) Mutation and Evaluation of Its Application

**DOI:** 10.1371/journal.pone.0090029

**Published:** 2014-02-28

**Authors:** Yongbin Zeng, Dezhong Li, Wei Wang, Mingkuan Su, Jinpiao Lin, Huijuan Chen, Ling Jiang, Jing Chen, Bin Yang, Qishui Ou

**Affiliations:** 1 First Clinical College, Fujian Medical University, Fuzhou, China; 2 Department of Laboratory Medicine, The First Affiliated Hospital of Fujian Medical University, Fuzhou, China; 3 Department of Laboratory Medicine, The Armed Police Hospital of Fujian, Fuzhou, China; 4 Center of Liver Diseases, The First Affiliated Hospital of Fujian Medical University, Fuzhou, China; Ghent University, Belgium

## Abstract

**Background:**

Long-term use of nucleos(t)ide analogues can increase risk of HBV drug-resistance mutations. The rtM204I (ATT coding for isoleucine) is one of the most important resistance mutation sites. Establishing a simple, rapid, reliable and highly sensitive assay to detect the resistant mutants as early as possible is of great clinical significance.

**Methods:**

Recombinant plasmids for HBV YMDD (tyrosine-methionine-aspartate-aspartate) and YIDD (tyrosine-isoleucine-aspartate-aspartate) were constructed by TA cloning. Real time allele specific locked nucleic acid quantitative PCR (RT-AS-LNA-qPCR) with SYBR Green I was established by LNA-modified primers and evaluated with standard recombinant plasmids, clinical templates (the clinical wild type and mutant HBV DNA mixture) and 102 serum samples from nucleos(t)ide analogues-experienced patients. The serum samples from a chronic hepatitis B (CHB) patient firstly received LMV mono therapy and then switched to LMV + ADV combined therapy were also dynamically analyzed for 10 times.

**Results:**

The linear range of the assay was between 1×10^9^ copies/μl and 1×10^2^ copies/μl. The low detection limit was 1×10^1^ copies/μl. Sensitivity of the assay were 10^−6^, 10^−4^ and 10^−2^ in the wild-type background of 1×10^9^ copies/μl, 1×10^7^ copies/μl and 1×10^5^ copies/μl, respectively. The sensitivity of the assay in detection of clinical samples was 0.03%. The complete coincidence rate between RT-AS-LNA-qPCR and direct sequencing was 91.2% (93/102), partial coincidence rate was 8.8% (9/102), and no complete discordance was observed. The two assays showed a high concordance (*Kappa* = 0.676, *P* = 0.000). Minor variants can be detected 18 weeks earlier than the rebound of HBV DNA load and alanine aminotransferase level.

**Conclusions:**

A rapid, cost-effective, high sensitive, specific and reliable method of RT-AS-LNA-qPCR with SYBR Green I for early and absolute quantification of HBV YIDD (ATT coding for isoleucine) variants was established, which can provide valuable information for clinical antiretroviral regimens.

## Introduction

Nucleos(t)ide analogues (NAs), such as Lamivudine (LMV), Telbivudine (LdT), Adefovir Dipivoxil (ADV) and Entecavir (ETV), are widely used for the treatment of chronic hepatitis B [1]. Unfortunately, long-term use of these drugs can increase risk of HBV drug-resistance mutations. The main site associated with resistance is mainly in the highly conserved tyrosine-methionine-aspartate-aspartate (YMDD) motif of the C domain of HBV polymerase. Methionine substituted by valine or isoleucine in codon 204 (i.e., rtM204V/I) were confirmed to confer resistance to LMV. The rtM204I can not only cause the selection of Lamivudine and Telbivudine resistance, but can also lead to Entecavir-resistance [2]. For many years, early detection of HBV drug-resistance mutants has received much attention due to its importance for the strategic treatment of chronic hepatitis B virus-infected patients.

Lots of highly sensitive detection technologies, such as next-generation sequencing, peptide nucleic acid-mediated PCR clamping, line probe assay (LiPA), liquid array, mass spectrometric analysis, have become available in recent years [3]–[7]. However, some limitations have also been found, i.e., special instruments may be present, testing costs may be high in some technologies, etc. Consequently, their wide application are somewhat restricted in clinical laboratories.

Although real time PCR such as allele-specific PCR (AS-PCR) for the detection of HBV mutants was reported years ago, little attention has been paid to AS-PCR using LNA-modified primers. Locked nucleic acid (LNA) is a nucleic acid analog with a 2-O, 4-C methylene bridge which generally increases its melting temperature. PCR primers modified with LNA nucleotides show very accurate mismatch discrimination and high specificity [8]. In the present study, we establish a novel assay named real time allele specific locked nucleic acid quantitative PCR (RT-AS-LNA-qPCR) with SYBR Green I for detection of HBV YIDD (ATT coding for isoleucine) mutation, and focus on its methodological evaluation and clinical application.

## Materials and Methods

### Patients

112 serum samples were obtained from chronic hepatitis B patients receiving nucleos(t)ide analogues (NAs) therapy in The First Affiliated Hospital of Fujian Medical University. 83 were male and 29 were female. The mean age was 38±13 years. Clinical data such as alanine aminotransferase (ALT) was measured. The study was in accordance with the approval of the ethics committee of The First Affiliated Hospital of Fujian Medical University and the ethical principles of the 1975 Declaration of Helsinki. Written informed consent was also obtained from each patient.

### Serum HBV DNA extraction

HBV DNA was extracted from serum samples using viral genomic DNA extraction kit (Beijing Xinnuo Company, China) according to the manufacturer's instruction and stored at −20°C until used.

### Primers for RT-AS-LNA-qPCR

Primers for RT-AS-LNA-qPCR were designed using Primer Premier 5.0 software (Premier Biosoft International, USA) according to the principle of AS-PCR technique. The design guidelines were provided by Exiqon (http://www.exiqon.com/oligo-tools). The polymorphism analysis of the most common genotypes (B and C) in China obtained from GenBank was performed on DNAMAN software (Lynnon BioSoft, Canada). The forward primer (KF) was at nucleotide positions 396∼416 for the amplification of both YMDD and YIDD (tyrosine-isoleucine-aspartate-aspartate). The reverse primers modified with a LNA at the 3′-end terminal position (L1) or penultimate position (L2) were designed to detect YIDD and YMDD, respectively. Wild type primer with last position of the nucleic acids lock (L2_l_) was synthesized. Non-degenerated primers including the forward (KF_n_) and the reverse (L1_n_ and L2_n_) with exact match to mutant and wild type plasmids were also included in this study (Table 1).

**Table 1:** Primers for RT-AS-LNA-qPCR.

Primer names	Sequences (5′-3′)	Positions (nt)	Length (bp)
KF	TCATMTTCCTCTKCATCCTGC	396∼416	21
L1	CCCCAAWACCACATCATC+A	741∼759	19
L2	CCCAAWACCACATCATC+CA	740∼758	19
L2_l_	CCCAAWACCACATCATC+C	740∼757	18
KF_n_	TCATCTTCCTCTGCATCCTGC	396∼416	21
L1_n_	CCCCAATACCACATCATC+A	741∼759	19
L2_n_	CCCAATACCACATCATC+CA	740∼758	19

KF/KF_n_ indicate the common forward primers to all reactions. L1/L2/L2_l_/L1_n_/L2_n_ indicate specific RT-AS-LNA-qPCR reverse primers. M indicates A/C; K indicates G/T; W indicates A/T; +A/+C indicate LNA nucleosides.

### Preparation of YMDD and YIDD plasmids for calibration curves

Two clinical samples harboring wild-type (YMDD, ATG coding for methionine) and mutant (YIDD, ATT coding for isoleucine) DNA sequences confirmed by direct sequencing were used for plasmids construction. Target DNA was amplified with primers (KF, L1 and L2) listed in Table 1. PCR was performed in a 25-μl reaction mixture containing 12.5 µl of 2× HotStart Taq PCR Mastermix (Tiangen, China), 0.7 µl of each primer (10 µM), 9.1 µl of ddH_2_O and 2.0 µl of DNA template. Thermal cycling conditions were as follows: initial denaturation at 94°C for 3 min, then 35 cycles with denaturation at 94°C for 30 s, annealing at 60°C for 30 s and extension at 72°C for 45 s, and a final elongation step at 72°C for 10 min. PCR products were electrophoresed on a 2.0% agarose gel and purified via PCR purification kit (Sangon, China), then cloned into pMD 18-T vector (Takara, Japan) and transformed into *E. coli* DH5α competent cell (Takara, Japan). The positive plasmids were sequenced and then isolated using Tiangen mini plasmid isolation kit (Tiangen, China). Plasmids were quantified by NanoDrop 2000 spectrophotometer (Thermo Fisher Scientific, USA). The corresponding copy number were calculated and 10-fold serially diluted from 1×10^10^ copies/μl to 1×10^1^ copies/μl using Easy Dilution Buffer (Takara, Japan) to generate standard concentrations.

### Preparation of different proportions of clinical mutant template

DNA from clinical sample harboring rtM204I confirmed by sequencing were mixed with wild-type clinical sample in different proportions. Specifically, clinical template containing 50% mutant HBV DNA was obtained by mixing 10 µl 1×10^5^ copies/μl clinical mutant DNA sample with 10 µl 1×10^5^ copies/μl clinical wild-type DNA sample, and clinical template containing 25% mutant HBV DNA was obtained by mixing 5 µl mutant DNA with 15 µl wild-type DNA. Clinical templates containing different proportions of mutant DNA (i.e., 20%, 10%, 5%, 1%, 0.5%, 0.05%, 0.04%, 0.03%, 0.02% and 0.01% of mutants) were also prepared in a similar way.

### RT-AS-LNA-qPCR

RT-AS-LNA-qPCR assay was performed on ABI 7500 Real-Time PCR system (Life Technologies, USA). The 25-μl PCR amplification reaction mixtures contained 12.5 µl of 2× SYBR Premix Ex Taq™ (Takara, Japan), 0.7 µl of each primer (10 µM), 0.5 µl of 50× ROX II reference dye (Takara, Japan), 8.6 µl of ddH_2_O and 2.0 µl of DNA template. Real time PCR conditions were: initial denaturation at 95°C for 30 s, followed by 40 cycles of denaturation at 95°C for 5 s, and annealing/extension at 60°C for 30 s. The post-amplification melting curve analysis was performed to confirm whether the nonspecific amplification was generated from primer-dimers.

### Direct Sequencing

To evaluate the performance of RT-AS-LNA-qPCR, all clinical samples were subjected to direct sequencing. The HBV DNA fragments containing the polymerase RT (reverse-transcriptase) domain were amplified using HBV sequencing kit (Shenyou, Shanghai) according to the manufacturer's instruction. Purified PCR products were sequenced with ABI 3130 genetic analyzer (Life Technologies, USA).

### Statistical analysis

Results obtained from RT-AS-LNA-qPCR were compared with those from sequencing. The statistical analysis was performed using statistical analysis software SPSS version 16.0 (SPSS Inc, USA) and GraphPad Prism software version 5.0 (GraphPad Software, USA). Complete concordance was considered if the results detected by RT-AS-LNA-qPCR and direct sequencing were identical. Partial concordance was considered if (1) RT-AS-LNA-qPCR provided additional information compared to that provided by sequencing, meaning that RT-AS-LNA-qPCR showed a mixture of wild-type and mutant sequences, whereas sequencing showed only one of the two results, or (2) sequencing showed a mixture of wild-type and mutant sequences but RT-AS-LNA-qPCR showed only a wild-type or a mutant sequence. Complete discordance was considered if one test showed a wild type and the other showed a mutant. Concordance was assessed by Cohen's *Kappa* test. A *P*<0.05 was considered statistically significant.

## Results

### Identification of recombinant plasmids by sequencing

The pMD-18-YMDD and pMD-18-YIDD recombinant clones were picked out and sequenced. The obtained DNA sequences were subjected to BLAST alignment against HBV genome database (http://www.ncbi.nlm.nih.gov/BLAST/). Blast results indicated the sequences were completely consistent with HBV reference genome. Therefore, the pMD-18-YMDD and pMD-18-YIDD recombinant plasmids were successfully constructed.

### Linear range and detection limit

**Figure 1:**
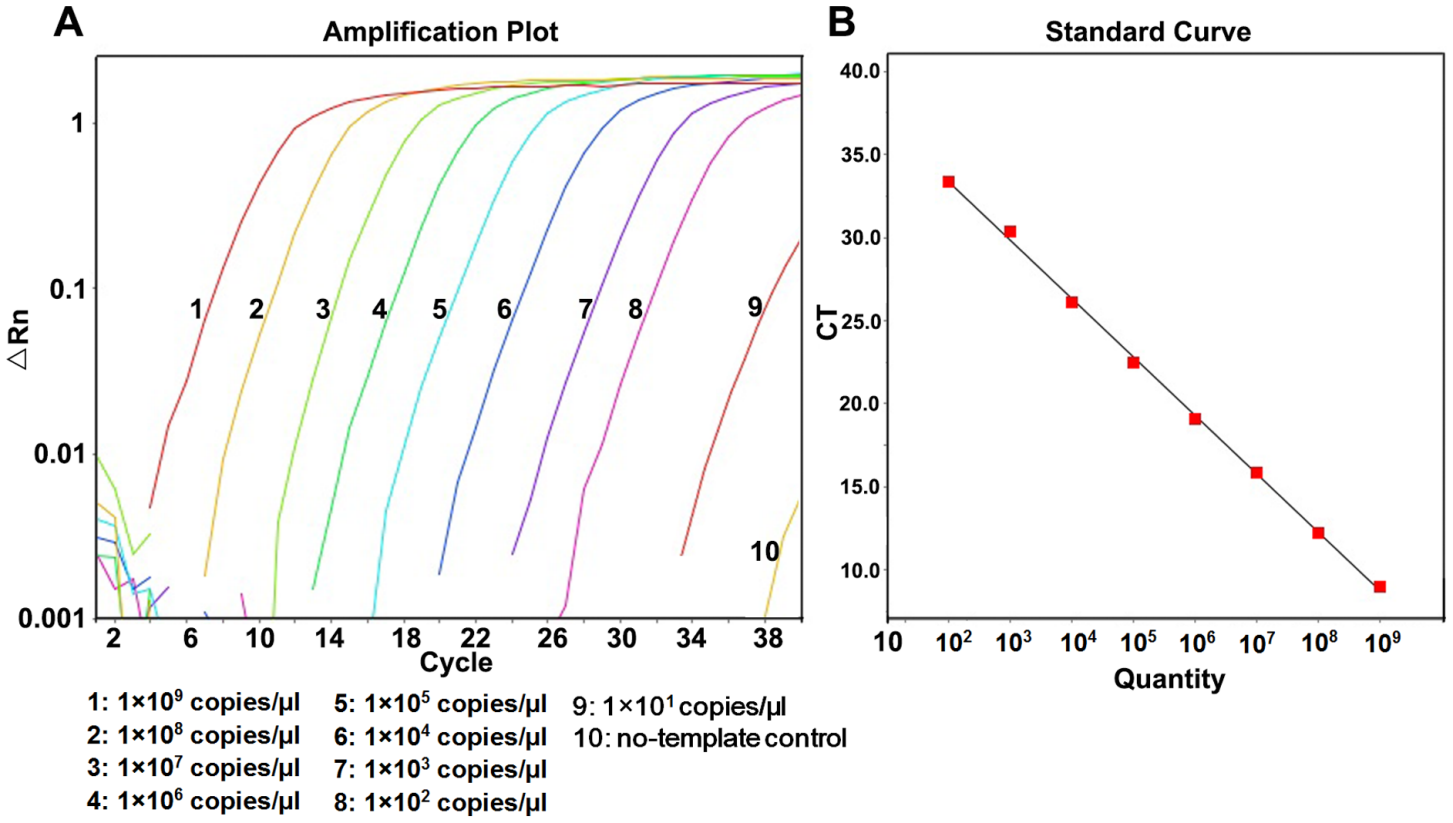
Amplification plot and standard curve of RT-AS-LNA-qPCR for pMD-18-YMDD. (A) Amplification plot with different colors represented different concentrations of pMD-18-YMDD plasmids which were illustrated in the figure. (B) Standard curve of pMD-18-YMDD: Y = -3.478X+41.654 (*R^2^* = 0.999).

**Figure 2:**
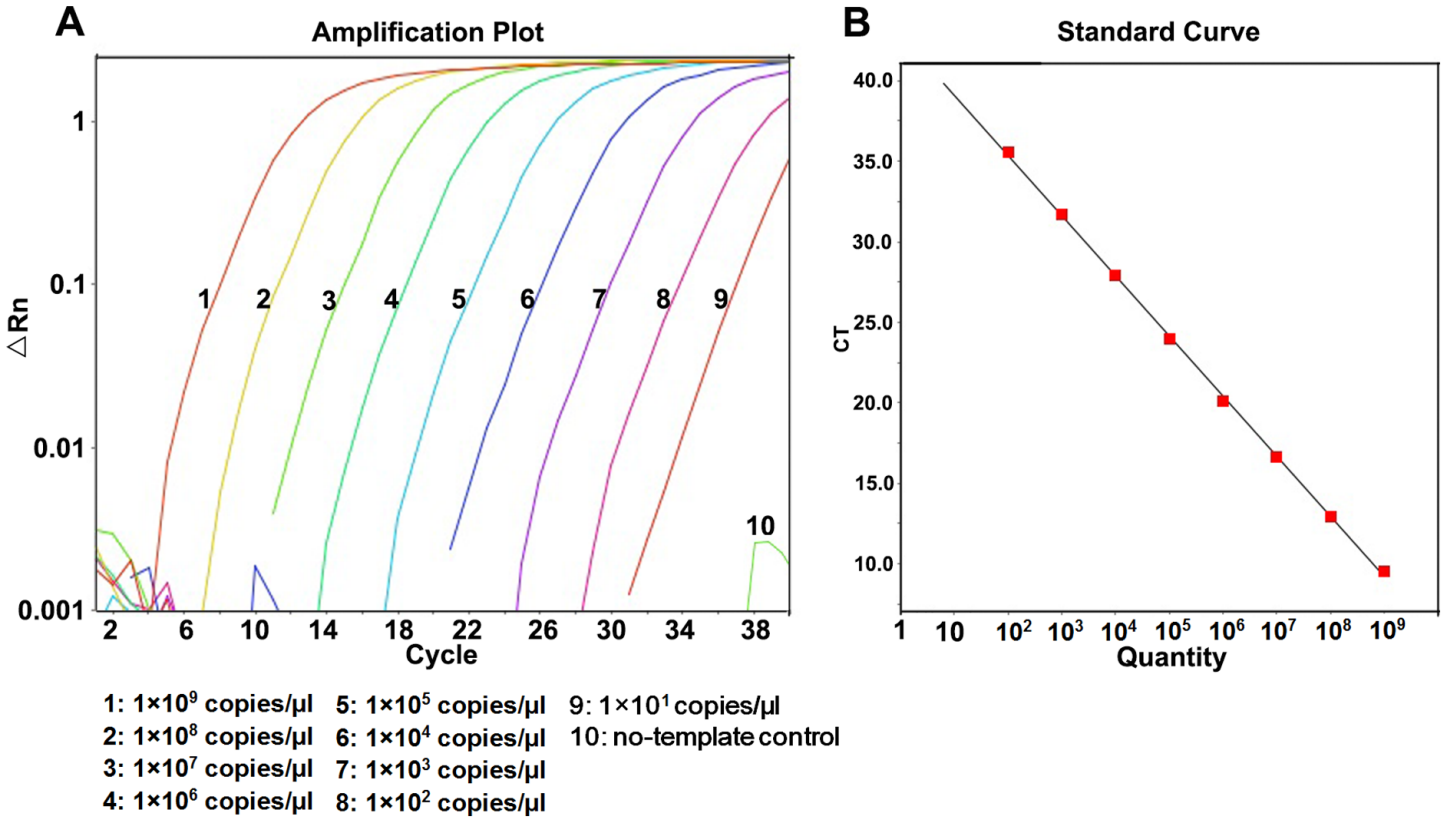
Amplification plot and standard curve of RT-AS-LNA-qPCR for pMD-18-YIDD. (A) Amplification plot with different colors represented different concentrations of pMD-18-YIDD plasmids which were illustrated in the figure. (B) Standard curve of pMD-18-YIDD: Y = −3.413X+37.793 (*R^2^* = 0.984).

1×10^10^ copies/μl∼1×10^1^ copies/μl recombinant plasmids were used to test the linear range and detection limit of RT-AS-LNA-qPCR (Figure 1 and Figure 2). There was an excellent linear correlation between the cycle number and the HBV DNA copy number from the concentration of 1×10^9^ copies/μl to 1×10^2^ copies/μl with correlation coefficients of 0.999 and 0.984 for the wild-type and mutant target sequences, respectively. The amplification efficiency were 94.9% and 96.3% for wild-type and mutant standard curves, respectively. Experiments using degenerated primers against each pure polymorphic templates (genotype B and C) were also done, and no differences were observed for the linear range and the detection limit (Figure S1)

### Specificity test

**Figure 3:**
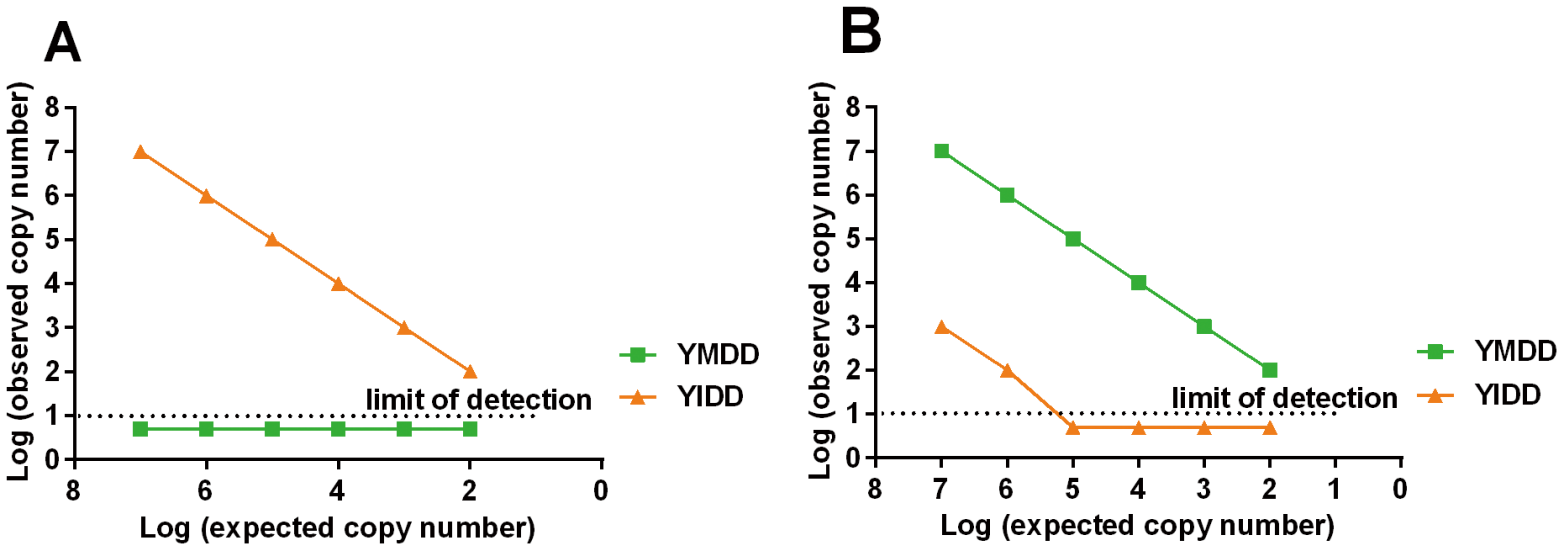
Cross-reactivity test of RT-AS-LNA-qPCR. (A) Specificity of mutant primer for detection of YMDD and YIDD plasmids. No nonspecific amplification was observed. (B) Specificity of wild-type primer for detection of YMDD and YIDD plasmids. Nonspecific amplification was detected at the concentration equal to or above 1×10^6^ copies/μl, but the corresponding mismatch products decrease significantly.

The cross-reactivity test was carried out using a dilution series of the template i.e., different concentrations of pMD-18-YMDD recombinant plasmids (1×10^7^ copies/μl∼1×10^2^ copies/μl) were amplified with mutant specific and wild-type specific primers, respectively, and so were the pMD-18-YIDD plasmids. As shown in Figure 3, no nonspecific amplification phenomenon was observed by mutant specific primers set (KF/L1), but, nonspecific amplification with the wild-type specific primers set (KF/L2) was detected at the concentration equal to or above 1×10^6^ copies/μl. However, the observed copy number was less than the expected copy number at least 4 logs. Interestingly, there was no nonspecific amplification observed when the mismatch template was below 1×10^6^ copies/μl. The priming efficiency of L2 and L2_l_ was compared and no difference was observed (Figure S2: The Ct values were found to be almost the same for primer L2 and primer L2_l_ when the same plasmid concentration was included in the test). The specificity of L2 was also compared with L2_l_; while 1×10^8^ copies/μl mutant DNA was added to the PCR reaction system, Ct (cycle threshold) values were 27.0 and 25.94 for L2 and L2_l_, respectively; when the template was 1×10^6^ copies/μl, the Ct values were 34.00 and 32.77 respectively. The Ct values were 36.97 and undetectable respectively, when mutant DNA was 1×10^5^ copies/μl. These results indicated the specificity of L2 was higher than L2_l_ ().

### Accuracy test

**Figure 4:**
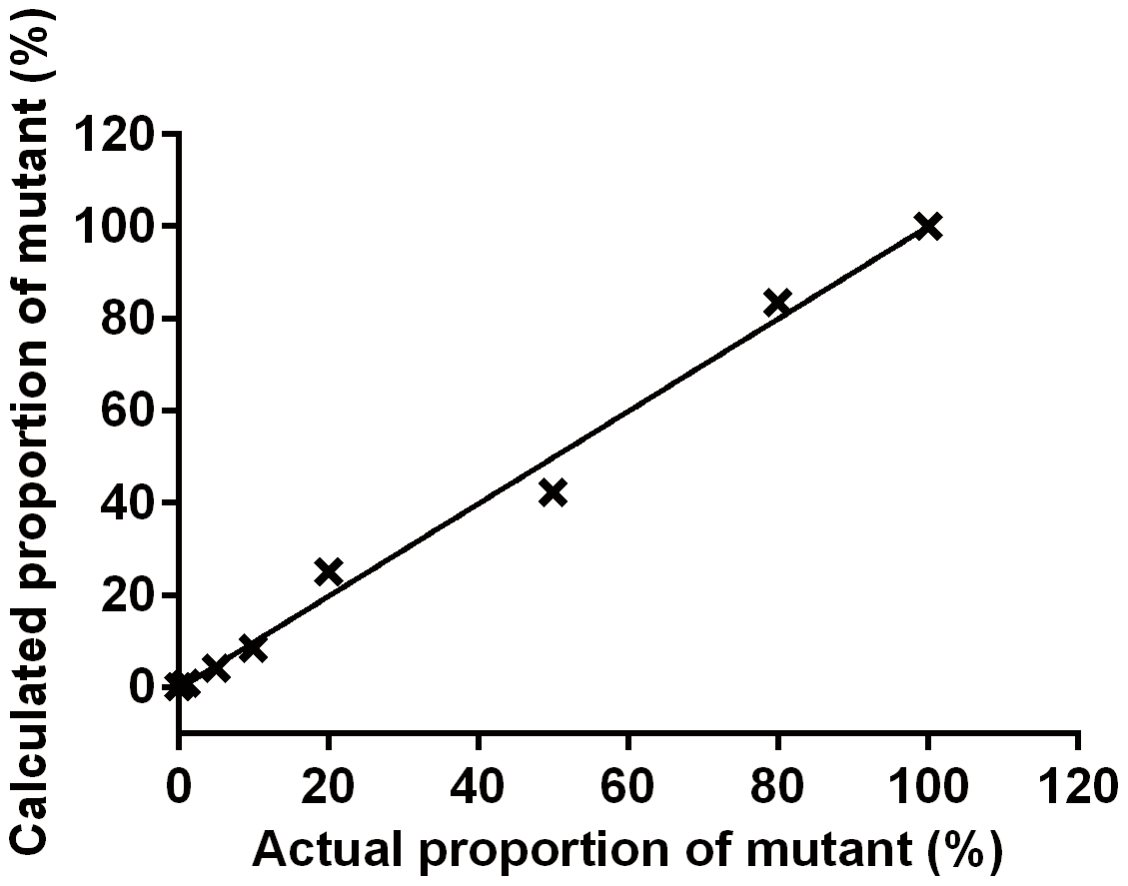
The correlation between actual and calculated proportion of mutant plasmid DNA at the concentration of 1×10^7^ copies/μl. *R^2^* = 0.9907, *P*<0.0001.

To assess the accuracy, a mixture of known quantity of wild-type and mutant standard plasmids were used to generate different proportions of mutant DNA, and both wild-type and mutant DNA were quantitatively analyzed by RT-AS-LNA-qPCR. As indicated in Figure 4, there was a significant linear correlation (*R^2^* = 0.9907, *P*<0.0001) between actual proportion of mutant and calculated proportion of mutant for HBV standard plasmid at the concentration of 1×10^7^ copies/μl.

### Reproducibility test

High (1×10^7^ copies/μl), medium (1×10^5^ copies/μl) and low (1×10^3^ copies/μl) concentrations of YMDD and YIDD recombinant plasmids were used as templates for 20 separate, simultaneous measurements by RT-AS-LNA-qPCR. The intra-run coefficient of variation (CV) was 0.74%, 0.91% and 1.07% for YMDD plasmids and 0.42%, 0.75% and 0.29% for YIDD plasmids. The quantitative assay was also done for 20 days consecutively. The inter-run CV was 1.12%, 1.79% and 1.93% for YMDD plasmids and 2.72%, 1.89%, 2.10% for YIDD plasmids.

### Sensitivity test

**Figure 5:**
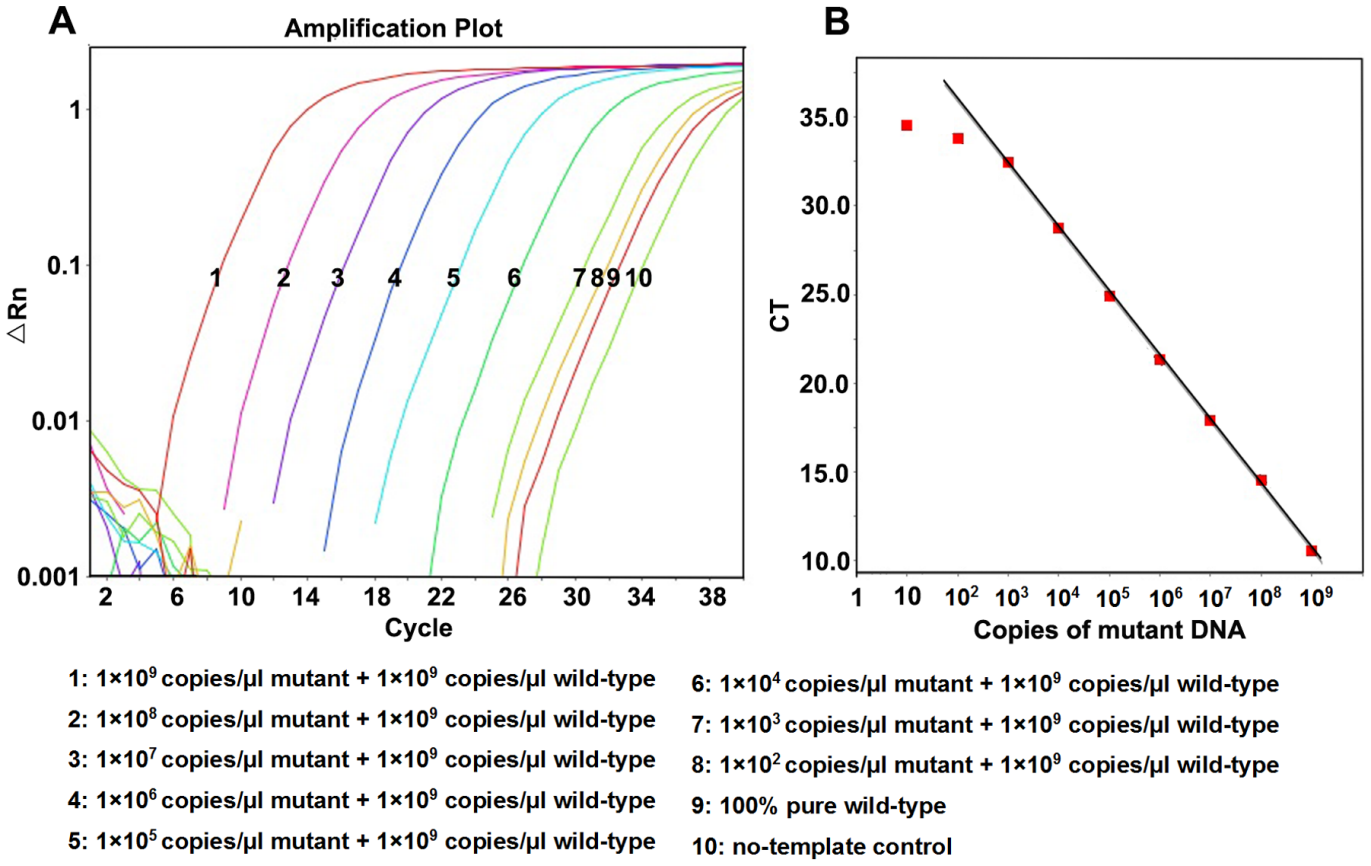
Sensitivity of RT-AS-LNA-qPCR in 1×10^9^ copies/μl wild-type DNA background. (A) Amplification plot with different colors represented different copies of mutant DNA balanced mixing with 1×10^9^ copies/μl wild-type DNA which were indicated in the figure. (B) Linear correlation diagram of sensitivity of RT-AS-LNA-qPCR in 1×10^9^ copies/μl wild-type DNA background.

A mixture of the dilution series of mutant plasmids with different fixed concentrations of wild-type DNA (1×10^9^ copies/μl, 1×10^7^ copies/μl and 1×10^5^ copies/μl, respectively) were tested with mutant primer to determine the minimum mutant DNA concentration at which RT-AS-LNA-qPCR could accurately and steadily quantify. The 100% pure wild-type DNA and no-template control were also tested. Results (Figure 5 and Figure S3) showed the assay could accurately and steadily detect 1×10^3^ copies/μl of mutant in the three different fixed concentrations of wild-type DNA, meaning that the sensitivity were 10^−6^, 10^−4^ and 10^−2^ in the wild-type background of 1×10^9^ copies/μl, 1×10^7^ copies/μl and 1×10^5^ copies/μl, respectively. Figure 5B showed it began to deviate from linearity when the concentration of mutant DNA was below 1×10^3^ copies/μl. The sensitivity test was also carried out using non-degenerated primers (KF_n_ and L1_n_) with exact match to mutant plasmids and there was no difference in detection sensitivity using the non-degenerated primers and the degenerated primers (Figure S4).

### Clinical utility and reproducibility

**Figure 6:**
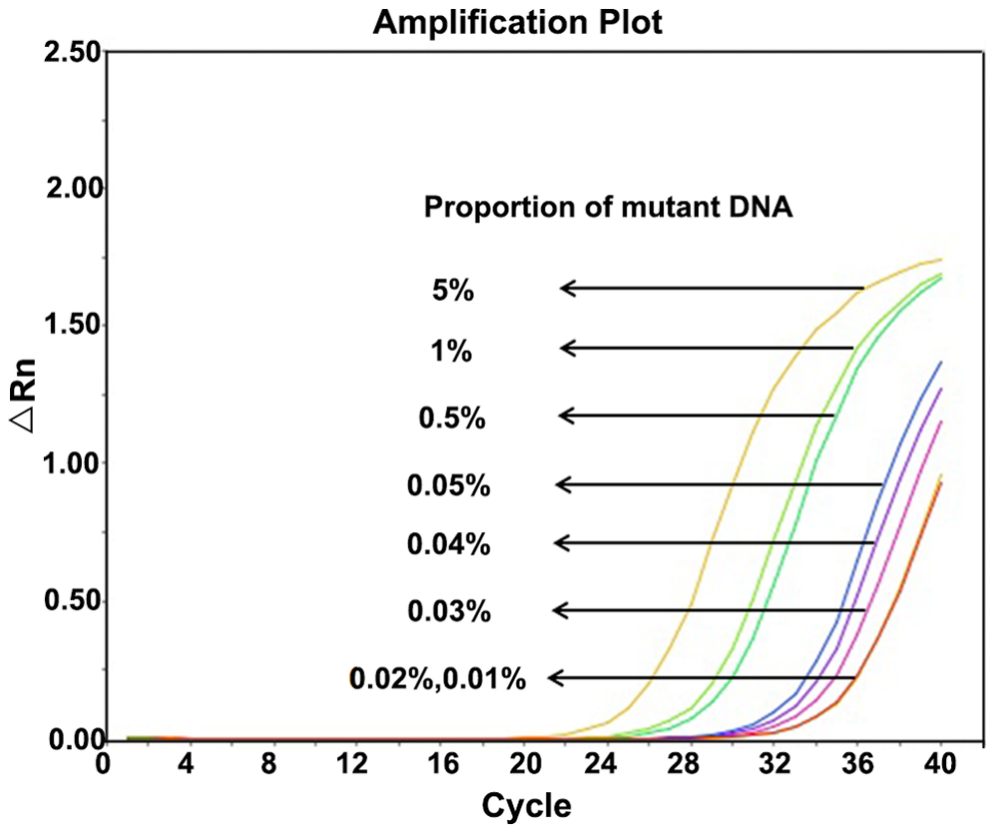
Clinical templates with different proportions of mutant DNA were tested by mutant specific primer set. Amplification plot with different colors represented different proportions of mutant DNA which were illustrated in the figure. The Ct of no-template control was undetectable.

The sensitivity was estimated using the different proportions of mutant above-mentioned (5%, 1%, 0.5%, 0.05%, 0.04%, 0.03%, 0.02% and 0.01%, respectively) by RT-AS-LNA-qPCR. We found that 0.03% of mutants could be accurately and steadily quantified by RT-AS-LNA-qPCR. Whereas the amplification curves of both 0.02% and 0.01% of mutants could not be discriminated from that of 0.03% of mutants (Figure 6). So 0.03% was regarded as the sensitivity of the assay of clinical samples with the most common polymorphisms.

To demonstrate reproducibility of quantification, the intra-run and inter-run reproducibility tests using clinical samples were also done. Repeated analysis demonstrated good reproducibility when the proportions of mutants was 0.03%.

### Sensitivity comparison of RT-AS-LNA-qPCR with sequencing analysis on clinical samples

Clinical samples containing different proportions (50%, 25%, 20%, 10%, 5%, 1%, 0.5%, 0.05%∼0.01%) of rtM204I were used to compare the sensitivity of RT-AS-LNA-qPCR with sequencing. As indicated in Table 2, RT-AS-LNA-qPCR could detect rtM204I at a proportion as low as 0.01% (detection limit) of the total population, whereas sequencing analysis only detect at a proportion of 10%.

**Table 2:** Detection sensitivity comparison of RT-AS-LNA-qPCR and sequencing on clinical samples containing different proportions of rtM204I variant.

Proportions of rtM204I	RT-AS-LNA-qPCR	Sequencing
50%	M/I	M/I
25%	M/I	M/I
20%	M/I	M/I
10%	M/I	M/I
5%	M/I	M
1%	M/I	-
0.5%	M/I	-
0.05%	M/I	-
0.04%	M/I	-
0.03%	M/I	-
0.02%	M/I	-
0.01%	M/I	-
NTC	N	N

M: Methionine; I: isoleucine; -: not performed; NTC: no-template control; N: undetectable.

### Comparison of RT-AS-LNA-qPCR with direct sequencing

**Figure 7:**
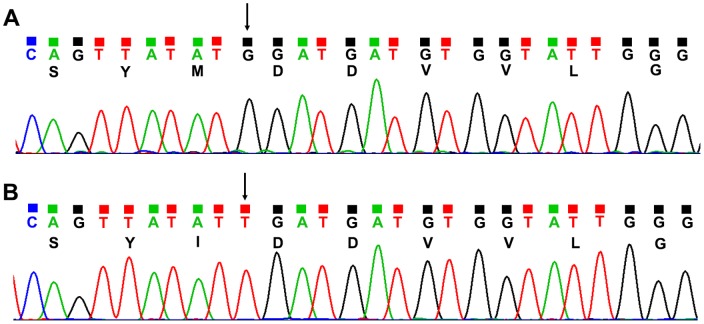
Typical chromatograms of the cloning sequencing of HBV rtM204 and rtM204I. (A) Chromatograms of rtM204 (wild-type). The bases in 204 site were ATG, indicated by the arrow. (B) Chromatograms of rtM204I (mutant). The bases in 204 site were ATT, indicated by the arrow.

102 NAs-experienced patients' sera were parallel analyzed by RT-AS-LNA-qPCR and direct sequencing. As shown in Table 3, among the 102 samples analyzed, 85 YMDD, 4 YIDD and 13 mixtures of YMDD + YIDD were detected by RT-AS-LNA-qPCR. By comparing the results obtained from RT-AS-LNA-qPCR with those from sequencing, the complete coincidence rate was 91.2% (93/102), partial coincidence rate was 8.8% (9/102), and no complete discordance was observed. Thus the two assays showed a high concordance (*Kappa* = 0.676, *P* = 0.000). Among the 6 partial concordant results (Only YIDD was detected by direct sequencing), the proportion of variants ranged from 80.8% to 99.8% of the total viral population was calculated by RT-AS-LNA-qPCR (Table 4). Among another 3 samples collected from CHB patients who were highly suspicious of drug resistance, only wild-type DNA was detected at rt204 by direct sequencing, however, both wild-type and mutant were detected by RT-AS-LNA-qPCR, and the proportion of mutant was only 8.4%, 9.8% and 9.5%, respectively, which was below the sensitivity of direct sequencing. 3 typical samples whose detected results were partially concordant between direct sequencing and RT-AS-LNA-qPCR were selected for cloning sequencing; and the established method is consistent with the cloning sequencing finding. The typical cloning sequencing chromatograms of HBV rt204 were showed in Figure 7.

**Table 3:** Concordance between RT-AS-LNA-qPCR and direct sequencing.

RT-AS-LNA-qPCR	Direct sequencing			Total
	YMDD	YMDD+YIDD	YIDD	
YMDD	**85**	0	0	85
YMDD+YIDD	*3*	**4**	*6*	13
YIDD	0	0	**4**	4
Total	88	4	10	102

Complete concordant results are shown in bold. Partial concordant results are shown in italics.

**Table 4:** Partial concordant results between RT-AS-LNA-qPCR and direct sequencing.

Patient number	Copy number of HBV DNA(copies/μl)		Mutant/Wild-type ratio	%Mutant/(Mutant+Wild-type)	Sequencing result	Motif
	Wild-type	Mutant				
2	9.32E+02	8.03E+04	86.2	98.9	ATT	YIDD
56	8.24E+05	3.47E+06	4.2	80.8	ATT	YIDD
11	5.25E+04	2.17E+07	413.3	99.8	ATT	YIDD
32	2.26E+04	1.13E+05	5.0	83.3	ATT	YIDD
7	3.31E+05	3.21E+06	9.7	90.7	ATT	YIDD
86	2.03E+04	1.13E+06	55.7	98.2	ATT	YIDD
22	3.47E+03	3.18E+02	0.092	8.4	ATG	YMDD
16	7.02E+04	7.63E+03	0.11	9.8	ATG	YMDD
65	1.22E+04	1.28E+03	0.10	9.5	ATG	YMDD

### Dynamic analysis of wild-type and mutant HBV DNA in a CHB patient by RT-AS-LNA-qPCR

**Figure 8:**
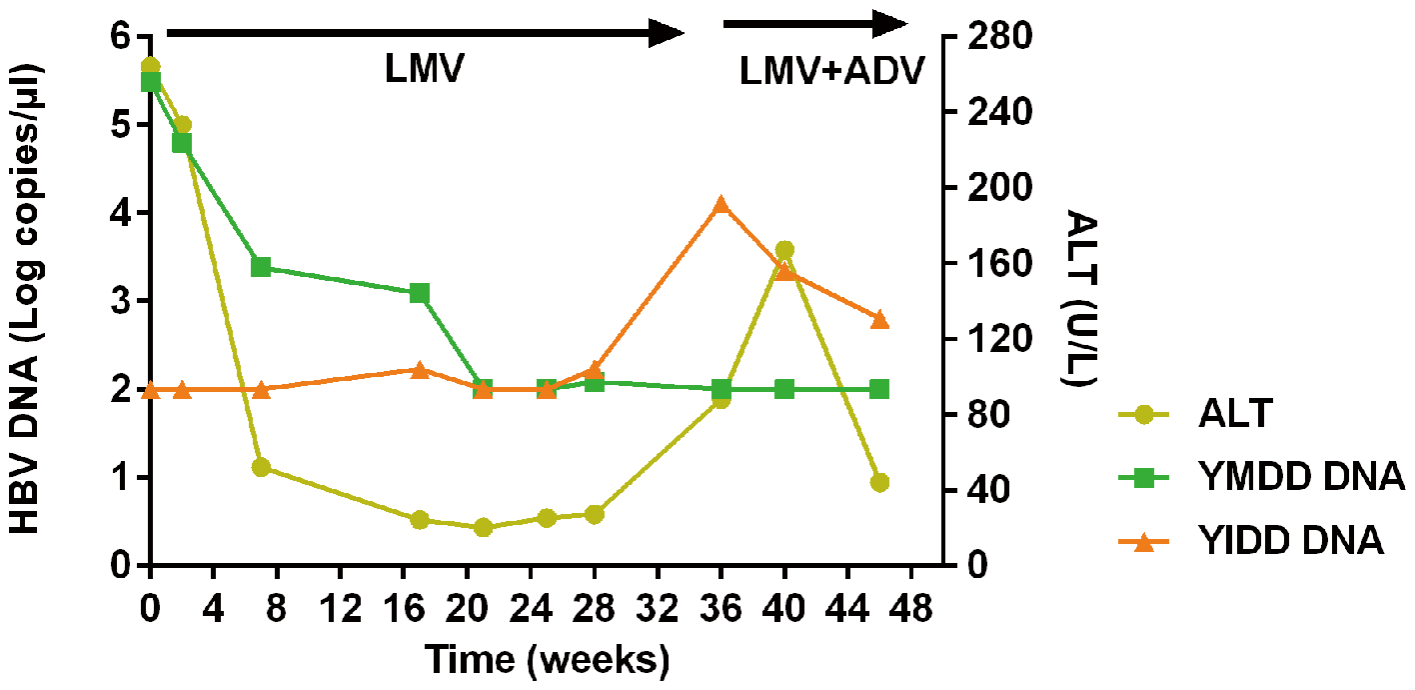
Dynamics of HBV YMDD, YIDD DNA levels and serum ALT level during LMV and LMV + ADV therapy in a CHB patient with breakthrough hepatitis.

A 48-week-follow-up study of dynamic of YMDD and YIDD HBV serum DNA levels and ALT levels during first LMV mono therapy and then LMV + ADV combined therapy was illustrated in Figure 8. As can be seen, HBV DNA level and ALT level declined substantially at the first 7 weeks and no YIDD variants were detectable at that time. However, at week 17, a minor fraction of YIDD variants had already been detected, accounting for 13.7%, 18 weeks before the rebound of HBV DNA load and alanine aminotransferase level. It was indicated that variants had become the predominant population with a percentage of 58.3% 11 weeks later. At week 36, YIDD variants population increased to 100%. ADV was added to ongoing lamivudine treatment at that time point. The bi-therapy decreased HBV DNA, however, very slowly; and variants remained the predominant population.

## Discussion

HBV drug resistant mutants are confirmed to exist in CHB patients prior to the initiating antiviral therapy with NAs. Previous researches have documented that the minor preexistence mutants can be gradually selected to become the dominant species under dual pressures of NAs and host immunity, and finally precede the occurrence of viral breakthrough or biochemical breakthrough [9]. Therefore, establishing a simple, rapid, reliable and highly sensitive assay to detect the resistant mutants as early as possible and analysis of their evolution during NAs-experienced are of great clinical significance.

A number of techniques regarding HBV mutants detection have been described. Direct sequencing, a qualitative assay, is considered to be the golden standard for HBV drug-resistance mutations detection, which is applied widely in clinical laboratories. But only mutations in circulating quasispecies pool present at>20% can be detectable [9]. Thus, it is not suitable for early detection of drug-resistance mutations. INNO-LiPA HBV DR, a commercial assay kit, has an analytical sensitivity between 5% and 10% and is much easier to perform and to automate [4], however, variants below 5% may be missed. In addition, it requires high cost and is only applied in developed countries. DNA chip is a high throughput, parallel and automatic technique and has become available in many fields, but it was not widely accepted in clinical laboratories due to its high cost. Ultradeep pyrosequencing (UDPS) can detect mutation of at least 1% of the total viral population, nonetheless, disadvantages have also been observed, including the short read length, high cost, complex data analysis and high error rate [3], [10]. Therefore, so far it has been used only for research purposes and not suitable for clinical use.

Allele-specific PCR, has also been termed amplification refractory mutation system PCR, selective PCR or mismatch PCR, which is a convenient and easily performed assay for mutation detection. However, it would be very important to reduce or to avoid false positive results by the optimization conditions. Basically speaking, primer design is the most important factor that would determine whether AS-PCR works specifically. There have been several studies with respect to the detection of HBV variants by AS-PCR [11]–[13], however, to our knowledge, few AS-PCR assays have been established taking advantage of LNA-modified primers and SYBR Green I.

Locked nucleic acid is a special nucleic acid analog with a 2-oxygen and 4-carbon atoms methylene bridge that locks the ribose group into the C3-endo conformation, thus, decreases ribosome structure flexibility and increases the phosphate backbone stability. It is generally accepted that LNA increases the melting DNA heteroduplex temperature between 1∼8°C per LNA nucleotide [8], [14].

To date, many reports have documented that primers at or near 3′-end modified with LNA nucleotides can be applied to improve the mismatch discrimination ability of allele-specific PCR assay [8], [14], [15]. But data about this application in HBV drug-resistance mutations detection are still scarce. Bhattacharya D et al. successfully developed an allele-specific quantitative PCR with combination of locked nucleic acid primers and a minor groove binder probe for the quantitative determination of minor viral quasispecies of the triple combination mutation rtV173L+rtL180M+rtM204V within one HBV genome. The assay could accurately detected 3×10^2^ copies of the triple mutant in the 3×10^8^ copies background of wild-type DNA [16].

Though genotypic detection of HBV resistance by AS-PCR using primers containing LNA bases has also been reported by Fang J et al. [17], several improvements were included in our study. There were some differences in primers. Firstly, ambiguity codes were introduced into the primers design because of the polymorphisms of HBV genomes, thereby allowing the primers to hybridize to different genotypes which are common in China. Different polymorphisms in the primer binding region may affect the sensitivity of mutant detection in the presence of the dominant wild type population. To validate detection sensitivity, experiments using non-degenerated primers with exact match to wild type and mutant plasmids were performed. The results indicated that there were no difference in sensitivity between the non-degenerated primers and degenerated primers. Degenerated primers against each pure polymorphic templates (genotype B and C) were also evaluated, whereas the detection sensitivity showed no difference as well. The likely explanations were as follows: On one hand, only two ambiguity codes were included in the forward primer design and only one in the reverse, consequently, the degeneracy is low. On the other hand, the positions of ambiguity codes were in the middle rather than 3′-end of the primers which may have limited influence on sensitivity test. Undoubtedly, the primers meet the degenerated primer design criteria. But the polymorphisms in HBV strains required the use of degenerated primer pairs according to a similar successful primer design described in Ntziora's research with respect to quantitative detection of the M204V hepatitis B virus minor variants [18]. Secondly, the LNA position of reverse primer specific for the wild-type sequence was at the penultimate position instead of 3′ termini which made it work more specifically. There were different opinions about positions of the nucleic acids locks in the literatures. And there was not a specific guideline on choosing the last position versus the penultimate position for incorporation of the LNA base so far. To a great extent, the position of LNA was determined empirically [16]. Most studies had shown a 3′ LNA residue in the primer at the SNP site (i.e., the mismatch site) could improve mismatch discrimination excellently. Therefore, the LNA nucleotide position in our primers was also chosen at the mismatch site. In a study of effect of locked nucleic acid (LNA) modification position upon representative DNA polymerase and exonuclease activities, Di Giusto et al. reported single LNA at the penultimate (L-2) nucleotide position generated nuclease resistance activity and provided improved discrimination [19]. And a similar successful primer design had also been adopted by Bhattacharya D et al. to quantify minority resistance variants in hepatitis B infection [16]. Inspired by their researches, we introduced an adenine (A) base into Primer L2 to allow LNA to locate at the penultimate to improve the specificity of Primer L2. And we compared the performance of L2 with L2_l_. It could be seen that the priming efficiency of L2 and L2_l_ were almost the same. When cross-reactivity tests were carried out, the specificity of L2 the was higher than L2_l_, as evidenced by an increase of the Ct value (ΔCt = CtL2 - CtL2_l_>1) while 1×10^8^∼1×10^6^ copies/μl mutant DNA was added to the PCR reaction system. Consequently, L2 exhibited an advantage over L2_l_ in this study. Beyond that, we also systematically evaluated its performance and clinical application. Besides plasmids, in the present work, clinical materials were used to estimate detection sensitivity and reproducibility of RT-AS-LNA-qPCR. The value of 0.03% was regarded as the sensitivity performed in the clinic. The results from RT-AS-LNA-qPCR and sequencing clearly demonstrated that the two assays showed a high concordance (*Kappa* = 0.676, *P* = 0.000). The complete concordance rate between the two assays was 91.2% (93/102). Among 6 partial results (YIDD was detectable by direct sequencing), the quantity of variants were represented 80.8% to 99.8%, a considerable amount of the total viral population. Among another 3 samples collected from CHB patients who were highly suspicious of drug resistance, only wild-type was detected at rt204 by direct sequencing, but both YMDD and YIDD were detectable by RT-AS-LNA-qPCR. The mutant ratio were 8.4%, 9.8% and 9.5%, respectively, below the sensitivity of direct sequencing. The results from cloning sequencing performed on 3 typical samples whose detected results were partially concordant between direct sequencing and RT-AS-LNA-qPCR were concordant with our established assay. These results indicated that the assay was reliable and sensitive enough to detect minor HBV variants.

Previous studies demonstrated that YMDD variants can be detected about 7 months before clinical breakthrough [7], although different reports differed [20]. In the dynamic observation of virological and biochemical characteristics of a LMV- resistant CHB infection patient, we found that the minor variants can be detected 18 weeks earlier than the rebound of HBV DNA and alanine aminotransferase level. So it may provide useful information for predicting breakthrough hepatitis and adjusting treatment strategies, however, further work focusing on larger-scale sample analysis of its evolution regularity are necessary during follow-up study.

Results obtained from our experimental data showed RT-AS-LNA-qPCR had a much higher analytical sensitivity for detecting minor variants in high wild type background and had several advantages over direct sequencing. Moreover, the assay is SYBR Green I -based quantitative real-time PCR, and there is no need to design target-specific probes, which is an inexpensive and easily extended method. In addition, instead of ΔΔCt calculation method, the assay uses standard curves in each run to absolute quantification wild type and mutant DNA amount, therefore, the accuracy of our assay is much more trustworthy.

Though RT-AS-LNA-qPCR showed excellent characteristics, some limitations were observed as well. Firstly, the specificity of wild-type primer is a little poor, the amplification of the mutant template with the wild-type primer occurred at a mutant viral concentration above 1×10^6^ copies/μl, but, the observed copy number was less than the expected copy number at least 4 logs. There were evidences that nonspecific priming phenomena caused by AS-PCR cannot be completely avoided [11]–[13].The different degrees of specificity between wild-type and mutant primers may be due to different primer-template mismatch types [21]. However, no nonspecific products occurred when mutant DNA level was below 1×10^6^ copies/μl. As the quantity of HBV DNA level is relatively low (below 1×10^6^ copies/μl) in patients with NAs treatment and two separate PCR including wild-type and mutant reaction tubes were run in parallel, the limitation can be ignored to some extent. Secondly, mutant primer was only designed for ATT, however, there are three codons coding for isoleucine (ATT, ATC and ATA). It should be noted that ATC and ATA were omitted in the present study due to their low frequency in China [5]. In a survey taken in our laboratory, we investigated 57 serum samples whose YIDD sequence were confirmed by DNA sequencing. Results showed that ATT accounted for 94.7% (54/57), while non-ATT only accounted for 5.3% (3/57) of total and appeared concurrently with ATT. The results were consistent with what has been previously reported by Sun [22] and Pyo Hong S [6]. So, the false negative rate was obviously very low. Thirdly, the limitation is that validation data was restricted to rtM204I. It is known that rtM204I is one of the most important and common resistance mutation sites, which can not only cause the selection of LMV and LdT resistance, but also lead to ETV resistance. Therefore, establishing a simple, rapid, reliable and highly sensitive assay to detect the rtM204I mutant as early as possible is of great clinical significance. In this study, we developed a new assay for the detection of the variant rtM204I with high-sensitivity. It provided a specific and reliable assay for detection of HBV YIDD variants, which is important for the management of CHB patients. Moreover, this assay may provide valuable reference for the detection of any other critical variants in the future study.

It remains unclear how quickly the minor variants will become the dominant and what mutant-to-wild-type ratio may cause treatment failure. In the follow-up study, we will combine with RT-ARMS-qPCR assay previously established in our laboratory to dynamic monitoring the evolution of HBV during NAs-experienced, so as to provide direct virological evidence for clinic.

In conclusion, a rapid, cost-effective, sensitive, specific and reliable method was developed for the quantification of the dynamic changes of HBV YIDD variants, which was a practical tool for HBV drug-resistant management. Further studies on methodological comparison including ultra-deep pyrosequencing, etc. will be included in our next study.

## Supporting Information

Figure S1 **Amplification plot of wild-type degenerated primers against each pure polymorphic templates (genotype B and C) for pMD-18-YMDD.** (A) Amplification plot with different colors represented different concentrations of pMD-18-YMDD plasmids of genotype B amplified with wild-type degenerated primers which were illustrated in the figure. (B) Amplification plot with different colors represented different concentrations of pMD-18-YMDD plasmids of genotype C amplified with wild-type degenerated primers which were illustrated in the figure.
(TIF)

Figure S2 **Efficiency and specificity test of L2 and L2_l_.** (A) Amplification plot with different colors represented different concentrations of pMD-18-YMDD plasmids amplified with L2 and L2_l_ primers, respectively, which were illustrated in the figure (curve 1 and 2: 1×10^5^ copies/μl; curve 3 and 4: 1×10^4^ copies/μl; curve 5 and 6: 1×10^3^ copies/μl; curve 7 and 8: 1×10^2^ copies/μl.). (B) Amplification plot with different colors represented different concentrations of pMD-18-YIDD plasmids amplified with L2 and L2_l_ primers, respectively, which were illustrated in the figure (curve 1 and 2: 1×10^8^ copies/μl; curve 3 and 4: 1×10^7^ copies/μl; curve 5 and 6: 1×10^6^ copies/μl; curve 7 and 8: 1×10^5^ copies/μl.).
(TIF)

Figure S3 **Sensitivity of RT-AS-LNA-qPCR in 1×10^7^ copies/μl and 1**×**10^5^ copies/μl wild-type DNA background.** (A) Amplification plot with different colors represented different copies of mutant DNA balanced mixing with 1×10^7^ copies/μl wild-type DNA which were indicated in the figure. (B) Amplification plot with different colors represented different copies of mutant DNA balanced mixing with 1×10^5^ copies/μl wild-type DNA which were indicated in the figure.
(TIF)

Figure S4 **Sensitivity detection of mutants in 1×10^9^ copies/μl wild-type DNA background using degenerated primer and non-degenerated primer.** (A) Amplification plot with different colors represented different copies of mutant DNA balanced mixing with 1×10^9^ copies/μl wild-type DNA, 100% pure wild-type DNA and no-template control respectively amplified with degenerated primer with exact match to mutant plasmid. (B) Amplification plot with different colors represented different copies of mutant DNA balanced mixing with 1×10^9^ copies/μl wild-type DNA, 100% pure wild-type DNA and no-template control respectively amplified with non-degenerated primer with exact match to mutant plasmid.
(TIF)
