# Supplementary figures and images for: Establishment of Real Time Allele Specific Locked Nucleic Acid Quantitative PCR for Detection of HBV YIDD (ATT) Mutation and Evaluation of Its Application
Source: PLoS One. 2014 Feb 28;9(2):e90029. doi: 10.1371/journal.pone.0090029 (PMC3938556; doi:10.1371/journal.pone.0090029)

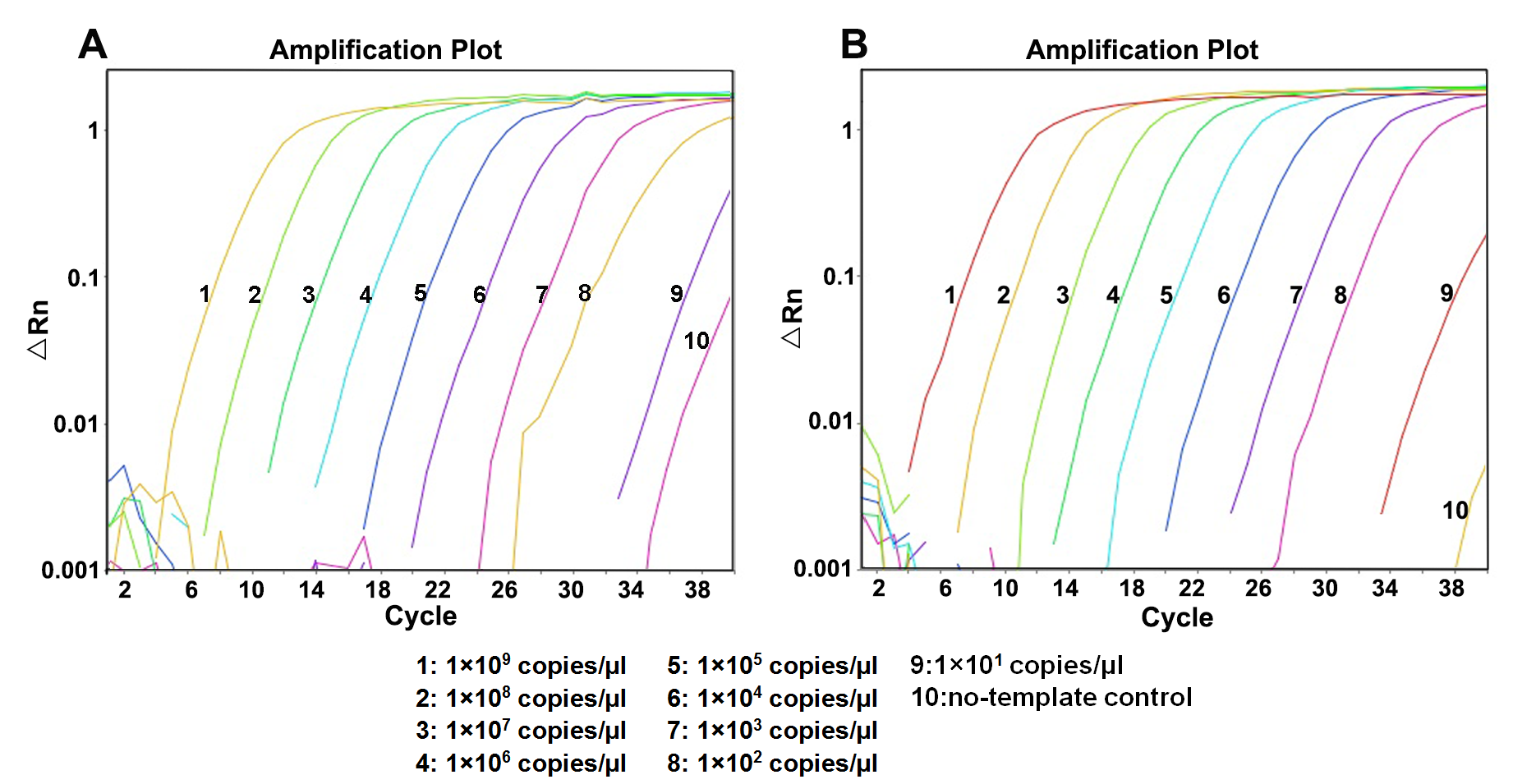

Supplement: Figure S1 — Amplification plot of wild-type degenerated primers against each pure polymorphic templates (genotype B and C) for pMD-18-YMDD. (A) Amplification plot with different colors represented different concentrations of pMD-18-YMDD plasmids of genotype B amplified with wild-type degenerated primers which were illustrated in the figure. (B) Amplification plot with different colors represented different concentrations of pMD-18-YMDD plasmids of genotype C amplified with wild-type degenerated primers which were illustrated in the figure. (TIF) [file pone.0090029.s001.tif]

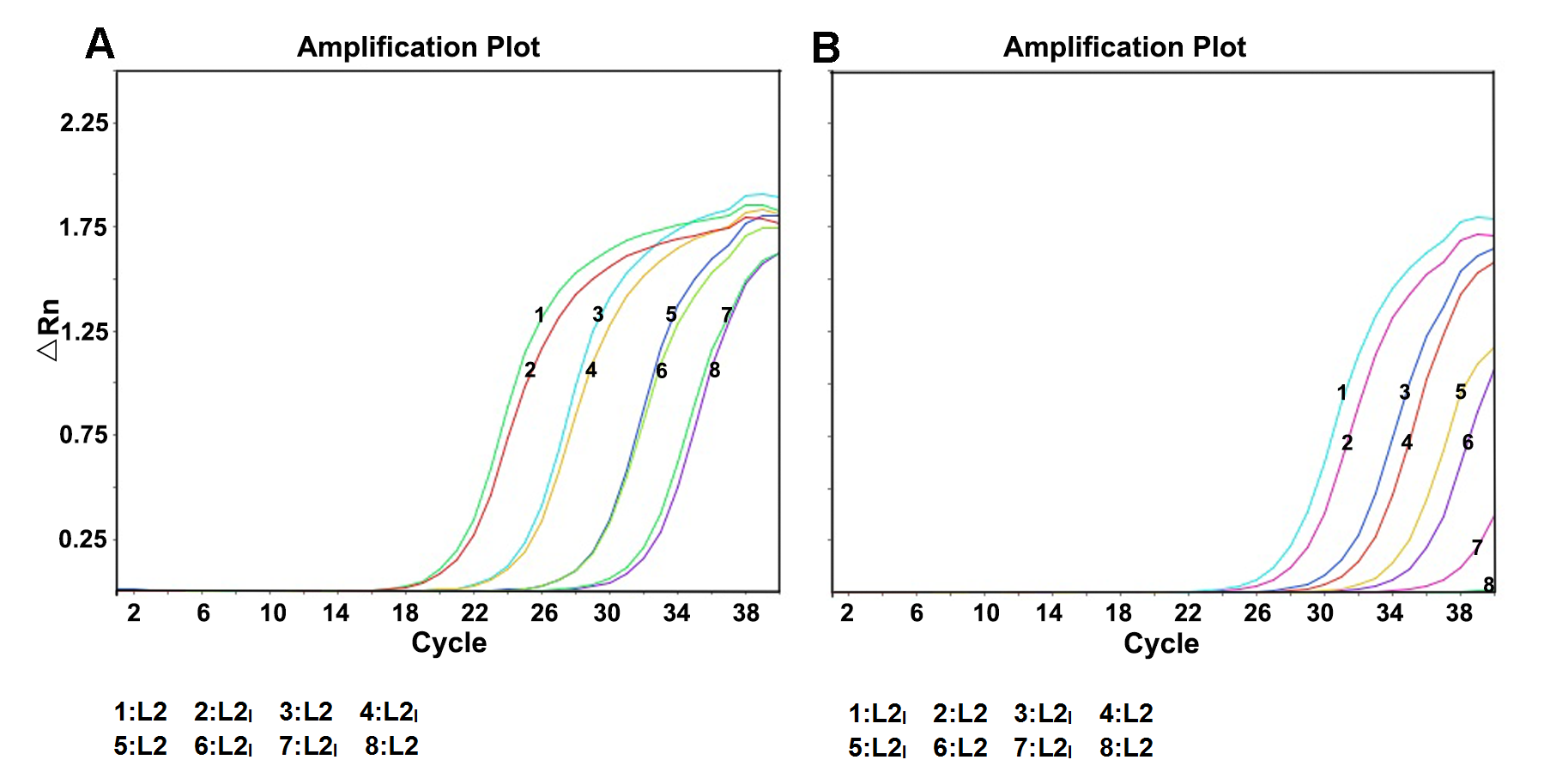

Supplement: Figure S2 — Efficiency and specificity test of L2 and L2l. (A) Amplification plot with different colors represented different concentrations of pMD-18-YMDD plasmids amplified with L2 and L2l primers, respectively, which were illustrated in the figure (curve 1 and 2: 1×10^5^ copies/μl; curve 3 and 4: 1×10^4^ copies/μl; curve 5 and 6: 1×10^3^ copies/μl; curve 7 and 8: 1×10^2^ copies/μl.). (B) Amplification plot with different colors represented different concentrations of pMD-18-YIDD plasmids amplified with L2 and L2l primers, respectively, which were illustrated in the figure (curve 1 and 2: 1×10^8^ copies/μl; curve 3 and 4: 1×10^7^ copies/μl; curve 5 and 6: 1×10^6^ copies/μl; curve 7 and 8: 1×10^5^ copies/μl.). (TIF) [file pone.0090029.s002.tif]

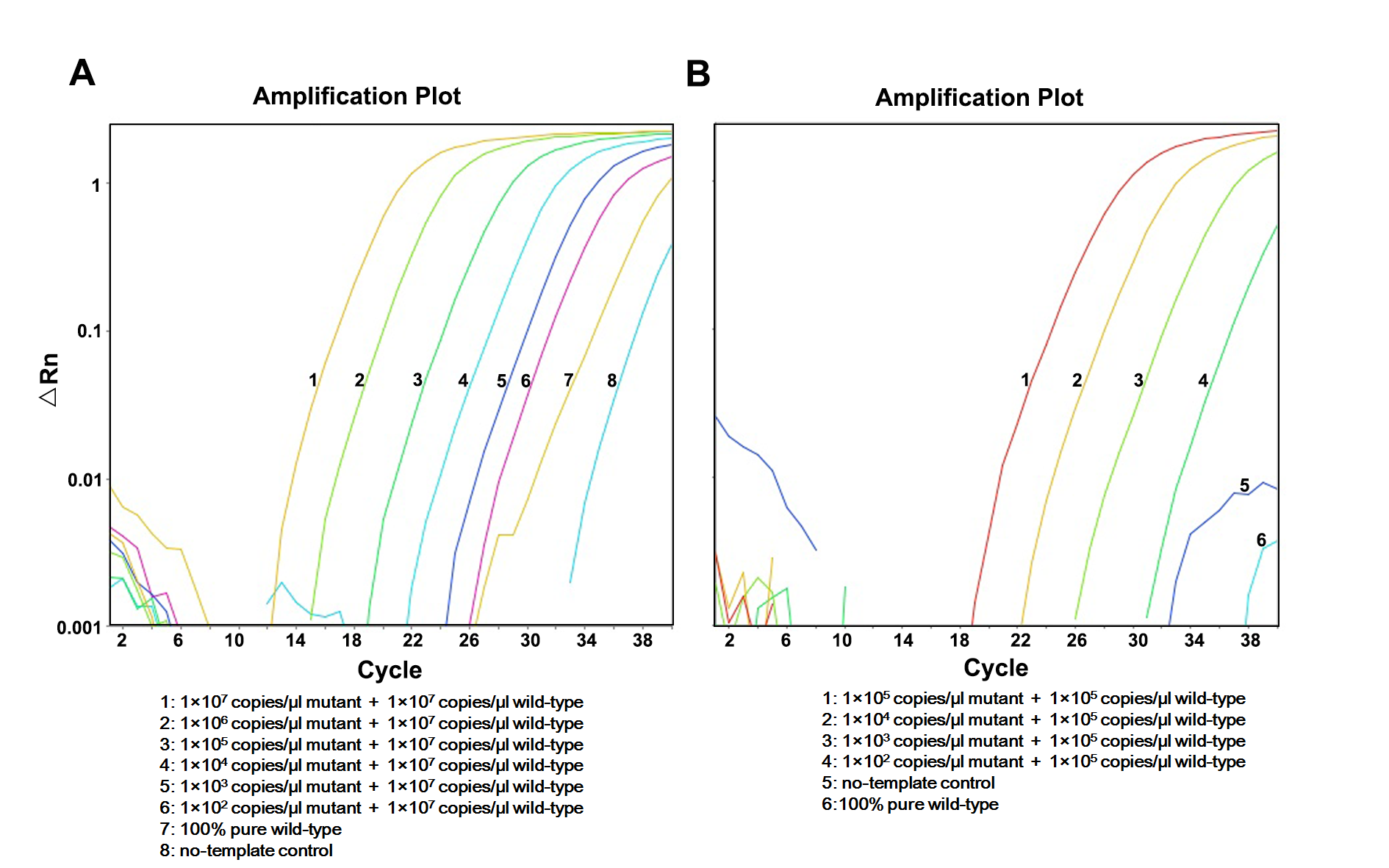

Supplement: Figure S3 — Sensitivity of RT-AS-LNA-qPCR in 1×10^7^ copies/μl and 1×10^5^ copies/μl wild-type DNA background. (A) Amplification plot with different colors represented different copies of mutant DNA balanced mixing with 1×10^7^ copies/μl wild-type DNA which were indicated in the figure. (B) Amplification plot with different colors represented different copies of mutant DNA balanced mixing with 1×10^5^ copies/μl wild-type DNA which were indicated in the figure. (TIF) [file pone.0090029.s003.tif]

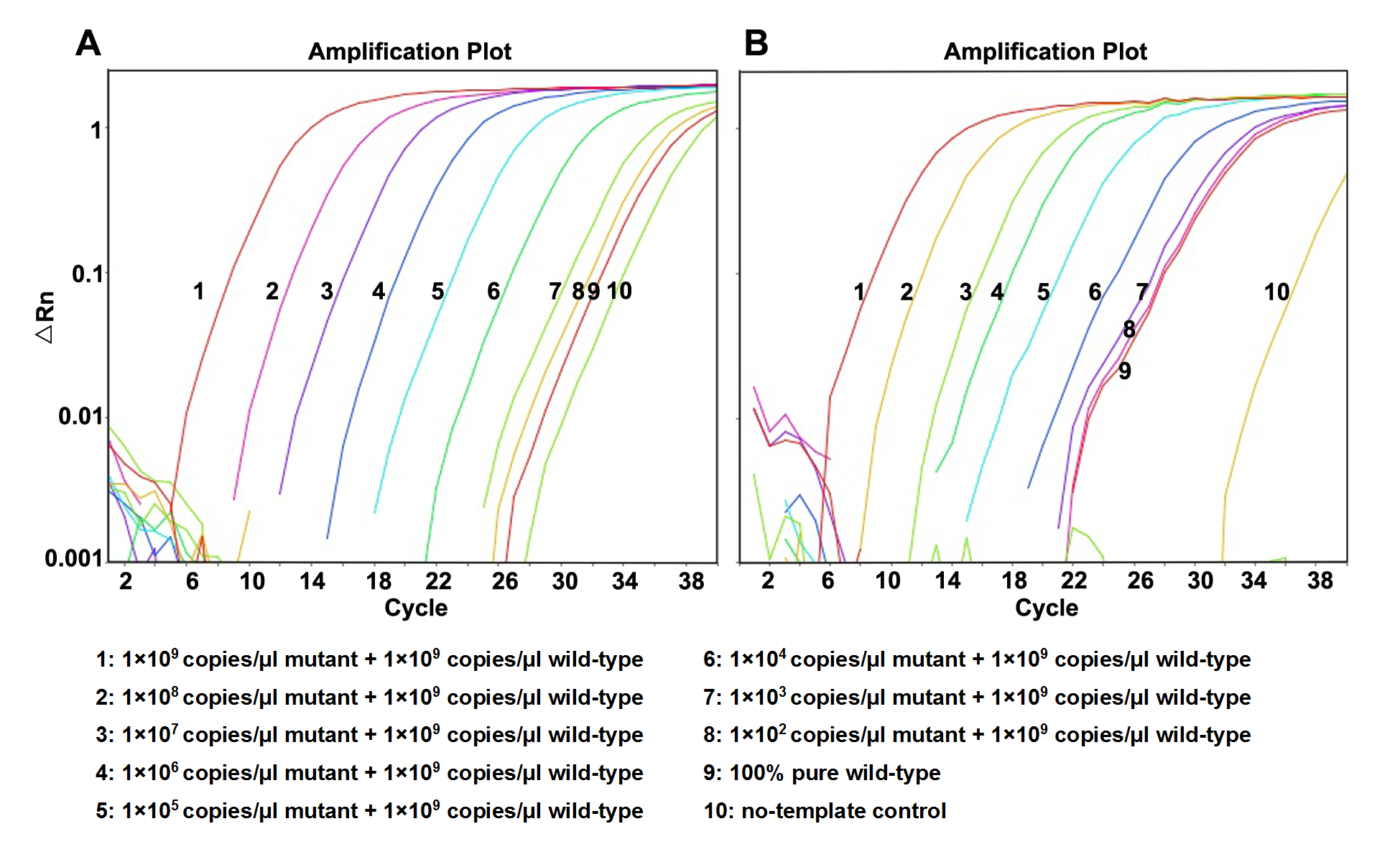

Supplement: Figure S4 — Sensitivity detection of mutants in 1×10^9^ copies/μl wild-type DNA background using degenerated primer and non-degenerated primer. (A) Amplification plot with different colors represented different copies of mutant DNA balanced mixing with 1×10^9^ copies/μl wild-type DNA, 100% pure wild-type DNA and no-template control respectively amplified with degenerated primer with exact match to mutant plasmid. (B) Amplification plot with different colors represented different copies of mutant DNA balanced mixing with 1×10^9^ copies/μl wild-type DNA, 100% pure wild-type DNA and no-template control respectively amplified with non-degenerated primer with exact match to mutant plasmid. (TIF) [file pone.0090029.s004.tif]
